# A Focal Adhesion-Related Gene Signature Predicts Prognosis in Glioma and Correlates With Radiation Response and Immune Microenvironment

**DOI:** 10.3389/fonc.2021.698278

**Published:** 2021-09-22

**Authors:** Haonan Li, Guohui Wang, Wenyan Wang, Jie Pan, Huandi Zhou, Xuetao Han, Linlin Su, Zhenghui Ma, Liubing Hou, Xiaoying Xue

**Affiliations:** ^1^Department of Radiotherapy, Second Hospital of Hebei Medical University, Shijiazhuang, China; ^2^Department of Central Laboratory, Second Hospital of Hebei Medical University, Shijiazhuang, China; ^3^Department of Radiation Oncology, PekingUniversity China-Japan Friendship School of Clinical Medicine, Beijing, China; ^4^The Department of Pathology, Stanford University School of Medicine, Stanford, CA, United States

**Keywords:** focal adhesion, glioma, prognosis, radiation response, tumor microenvironment, immune checkpoints

## Abstract

**Background:**

Glioma is the most frequent brain malignancy presenting very poor prognosis and high recurrence rate. Focal adhesion complexes play pivotal roles in cell migration and act as hubs of several signaling pathways.

**Methods:**

We used bioinformatic databases (CGGA, TCGA, and GEO) and identified a focal adhesion-related differential gene expression (FADG) signature by uniCox and LASSO regression analysis. We calculated the risk score of every patient using the regression coefficient value and expression of each gene. Survival analysis, receiver operating characteristic curve (ROC), principal component analysis (PCA), and stratified analysis were used to validate the FADG signature. Then, we conducted GSEA to identify the signaling pathways related to the FADG signature. Correlation analysis of risk scores between the immune checkpoint was performed. In addition, the correlation of risk scores and genes related with DNA repair was performed. CIBERSORT and ssGSEA were used to explore the tumor microenvironment (TME). A nomogram that involved our FADG signature was also constructed.

**Results:**

In total, 1,726 (528 patients diagnosed with WHO II, 591 WHO III, and 603 WHO IV) cases and 23 normal samples were included in our study. We identified 29 prognosis-related genes in the LASSO analysis and constructed an eight FADG signature. The results from the survival analysis, stratified analysis, ROC curve, and univariate and multivariate regression analysis revealed that the prognosis of the high-risk group was significantly worse than the low-risk group. Correlation analysis between risk score and genes that related with DNA repair showed that the risk score was positively related with *BRCA1*, *BRCA2*, *RAD51*, *TGFB1*, and *TP53*. Besides, we found that the signature could predict the prognosis of patients who received radiation therapy. SsGSEA indicated that the high-risk score was positively correlated with the ESTIMATE, immune, and stromal scores but negatively correlated with tumor purity. Notably, patients in the high-risk group had a high infiltration of immunocytes. The correlation analysis revealed that the risk score was positively correlated with *B7-H3*, *CTLA4*, *LAG3*, *PD-L1*, and *TIM3* but inversely correlated with *PD-1*.

**Conclusion:**

The FADG signature we constructed could provide a sensitive prognostic model for patients with glioma and contribute to improve immunotherapy management guidelines.

## Introduction

Glioma account for the largest proportion of malignant craniocerebral tumors (1). The currently available treatments include total resection, radiotherapy, and chemotherapy. The National Comprehensive Cancer Network (NCCN) guideline recommends chemoradiation +/- tumor treatment fields (TTFs) for adjuvant treatment in primary diagnosed GBMs. Clinical cancer studies have advanced in recent years, although the overall survival (OS) has not improved (2). Patients with high-grade glioma who receive concurrent temozolomide and postoperative radiation achieve a median survival of only 14.6 months. Meanwhile, patients receiving radiotherapy alone achieve a median survival of only 12.1 months. Glioma are highly aggressive and have high fatality rates (3). Glioma has been classified into four grades by the World Health Organization (WHO) 2016 Classification of glioma. Low-grade glioma (WHO II) includes oligodendrogliomas and astrocytomas, while WHO III includes anaplastic oligodendrogliomas, anaplastic astrocytomas, anaplastic oligoastrocytomas, and anaplastic ependymomas. Glioblastoma (GBM) has been defined as high-grade glioma, which is resistant to chemoradiotherapy (4). Immunotherapy is an emerging method of treatment for several kinds of cancer, such as breast cancer, lung cancer, and prostate cancer (5, 6). However, there is no efficient treatment for progressive disease in glioma and there is an urgent need for efficient management strategies.

Focal adhesion involves multiprotein complexes that act to anchor the actin cytoskeleton to the extracellular matrix (ECM). These complexes consist of integrins and actins, structural proteins including vinculin, talins, and signaling molecules such as focal adhesion kinase (7). In cancer cells, adhesion to the ECM may mediate radioresistance, chemotherapy, and resistance to targeted therapy (8). Focal adhesions can interact with the stroma signaling pathways and cooperate with downstream targets of integrins and growth factor receptors. These complexes play a pivotal role in cell survival, proliferation, differentiation, and invasion. Integrins are catalytically inactive receptors, which combine with the ECM directly and activate downstream signal transduction (9). Integrins promote a special type of EMT in glioma that endows cancer cells with the ability of metastasis (10). The conformational memory of integrin reinforces the assembly of focal adhesions and induces cell migration (11). Inhibition of the focal adhesion signaling pathway may be a promising therapeutic target for gliomas.

In this study, we mined the online databases including the Gene Expression Omnibus (GEO), The Cancer Genome Atlas (TCGA), and the Chinese Glioma Genome Atlas (CGGA), and constructed a focal adhesion-related differential gene expression (FADG) signature in glioma. We found that the FADG signature was associated with prognosis, radiation response, and the immune microenvironment, and especially with immune cell infiltration. Our results revealed that this FADGs signature could accurately predict the prognosis and provide precise guidelines for the treatment of glioma.

## Materials and Methods

### Dataset Retrieval

Data from a total of 180 samples from glioma patients including 23 normal samples and 157 cases were obtained from the Gene Expression Omnibus database, expression profiling arrays (dataset GSE4290), and platform GPL570 (https://www.ncbi.nlm.nih.gov/). In total, 1,018 mRNA sequences from glioma patient samples and their corresponding clinical information were collected from the CGGA database as the training and internal cohort (http://www.cgga.org.cn/index.jsp). RNA sequence and clinical data including 449 LGG and 143 GBM from the TCGA database as the external validation cohort. Overall, 199 focal adhesion genes were retrieved from the MSigDB database (https://www.gsea-msigdb.org/gsea/msigdb).

### Identification of Focal Adhesion-Related Differential Expression Genes

The GSE4290 database was mined to identify the differentially expressed genes (DEGs), which were then analyzed by the R software 4.0.0. setting a gene expression threshold of |log fold change (FC)| > 1 and false discovery rate (FDR) < 0.05. FADGs were filtered, which overlapped with the focal adhesion-relevant genes and DEGs. A Protein-protein interacting (PPI) network of these candidate genes was constructed using STRING (https://www.string-db.org/). These candidate genes underwent univariate regression analysis in R using the CGGA data including RNA sequencing, OS, and living status data.

### Construction of the Focal Adhesion-Relevant Signature

Patient data from the CGGA were randomly divided into two cohorts: a training cohort and a testing cohort. Next, the least absolute shrinkage and selection operator (LASSO) regression analysis was performed to remove collinearity among these genes. DEGs and survival probability associated of these genes were analyzed using the GEPIA database (http://gepia.cancer-pku.cn/). Immunohistochemistry images were obtained from the Human Protein Atlas (HPA) database (https://www.proteinatlas.org/). A prognosis-related signature was conducted based on the expression of the candidate genes and regression analysis coefficient values. The algorithm was as follows:


$$riskscore=\sum_{i=1}^{n}\left(Coefi*Xi\right)$$

A patient risk score was calculated using this formula, and was used to stratify patients according to the median risk score into low- and high-risk groups. ‘SurvivalROC’ R package, Harrell’s concordance index to assess the predictive value of the FADGs signature for prognosis.

### Validation of the Focal Adhesion-Related Differential Genes Signature

The training cohort and testing cohorts from the CGGA and TCGA validation cohorts were used for the following analysis. Missing clinical data were eliminated. ‘Survival’ and ‘survminer’ packages were utilized in R to perform a survival probability between the high- and low-risk groups. The ‘SurvivalROC’ package was utilized to perform receiver operating characteristic (ROC) curve analysis to verify the accuracy of the model for the 1-, 3-, and 5-year survival. Survival risk was plotted by R using the ‘riskpot’ package. Next, ‘scatterplot3d’ was used to perform principal component analysis (PCA). Stratification analysis of the TCGA and CGGA datasets was plotted in R for the high- and low-risk groups. Multivariate and univariate Cox regression analysis was plotted for both the training and testing cohorts. Harrell’s concordance index was also programmed.

### GSEA Was Performed to Identify the Involved Gene Pathways

Hallmark, Gene Ontology (GO), and Kyoto Encyclopedia of Genes and Genomes (KEGG) analysis between the low- and high-risk groups was conducted using GSEA_4.1.0. to explore the functional annotation of the DEGs and for the systematic analysis of the gene functions (h.all.v7.2.symbols, c2.cp.kegg.v7.2.symbols, c5.go.v7.2.symbols). The results were filtered by Normalized Enrichment Score (NES) > 1 and FDR q-val < 0.05.

### Correlation Analysis of Focal Adhesion-Related Differential Gene Signature and Radiation Response

Correlation analysis between genes that related with DNA repair and risk score were programmed using the training and testing cohorts in the CGGA database. Radiation response of patients after radiation therapy were extracted from the TCGA cohort. Differential analysis of the risk scores of complete remission (CR) and progression disease (PD) were analyzed.

### Correlation Analysis of Focal Adhesion-Related Differential Gene Signature and Immune Cell Infiltration

Single-sample gene set enrichment analysis (ssGSEA) is an algorithm used to evaluate the level of immune cell infiltration in a single sample according to the expression levels of immune cell-specific markers. Patients in the CGGA training cohort and testing cohort were imported for the ssGSEA analysis. Patients with glioma were analyzed by R using the ‘limma’, ‘GSVA’, and ‘GSEABase’ packages. Next, samples with risk scores were imported into ESTIMATE, for the Estimation of Stromal and Immune cells in malignant tumor tissues using Expression data analysis, to verify the ssGSEA results. We performed a correlation analysis between the risk score and ESTIMATE, stromal, and immune scores, and tumor purity. Next, the correlation between the risk score and expression of immune check point was analyzed. CIBERSORT was performed to analyze the 22 distinct leukocyte subsets in the tumors based on bulk transcriptome data to detect the tumor purity and to explore the TME using the ‘e1071’ and ‘parallel’ packages.

### Establishment of the Nomogram

A nomogram was constructed using the CGGA cohort to predict the prognosis of patients combining the clinical features and risk scores to assess the accuracy of the model. A calibration curve was generated to evaluate the accuracy of the nomogram. To demonstrate the incremental value of the FADGs signature over the clinicopathological characteristic for an individualized assessment of the OS, the decision curve was constructed.

### Statistical Analysis

R software version 4.0.0 (Statistics Department of the University of Auckland) with corresponding packages 160 and Graphpad Prism, version 7 (GraphPad Software,San Diego, California USA) were used for statistical analyses. A P-value < 0.05 was considered statistically significant.

## Results

### Analysis of Differentially Expressed Genes in Glioma Patients Correlated With Focal Adhesion

**Figure 1:**
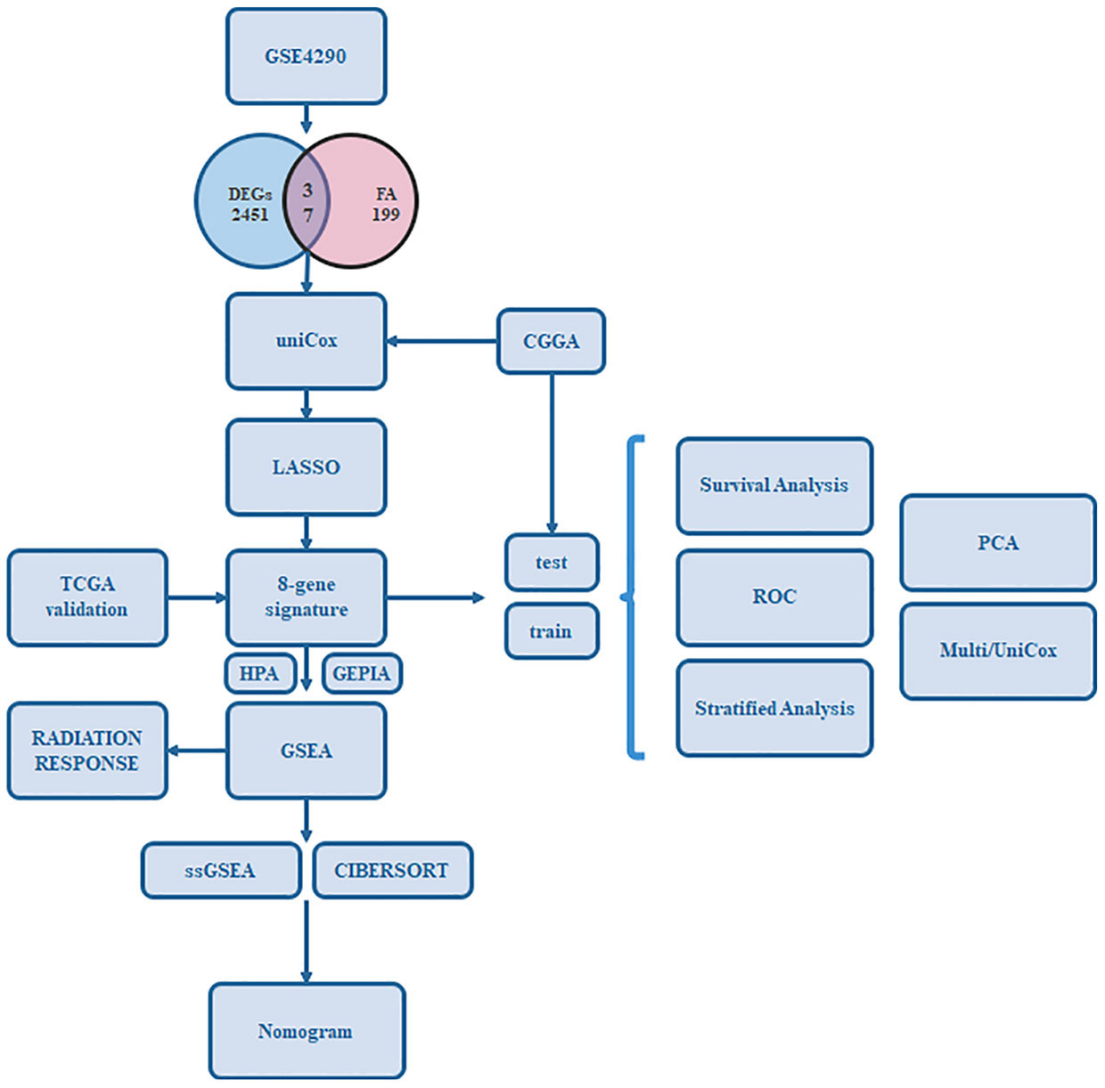
Flow chart of our study.

**Figure 2:**
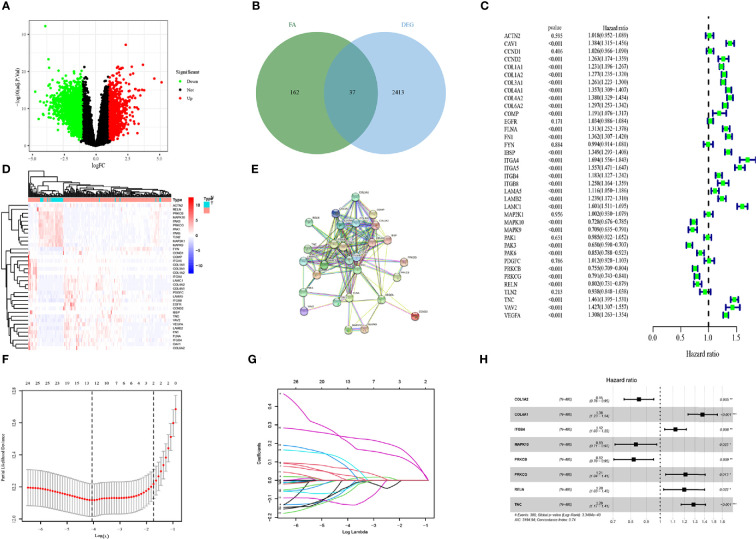
Identification of candidate genes and LASSO-COX analysis **(A)** Volcano plot of differentially expressed genes in glioma from the GEO database. **(B)** Heatmap of differentially expressed genes in glioma from the GEO database. **(C)** Univariate regression analysis of FADGs. **(D)** Venn plot of differentially expressed genes and focal adhesion related genes. **(E)** Protein-protein interacting network. **(F, G)** Construction and validation of focal adhesion related signature. **(H)** Multivariate cox regression analysis of eight candidate genes.

A flow chart to illustrate the workflow used in our study is shown in **Figure 1**. The features of patients enrolled in this study are listed in **Table 1**. To identify the DEGs, the GSE4290 dataset from the GEO database was extracted. There was a total of 2,450 DEGs between glioma patients and normal brain tissues. Genes covering 1,450 upregulated and 1,425 downregulated were plotted in a volcano plot (**Figure 2A**). A total of 37 FADGs were selected from the overfitting group of 2,450 genes and 199 focal adhesion relevant genes (**Figure 2B**). Then, univariate regression analysis was performed and 29 genes associated with the prognosis were selected as candidate genes filtering with a threshold of P < 0.05 (**Figure 2C**). A heatmap of gene expression for each patient was plotted (**Figure 2D**). A PPI network was constructed involving the 37 FADGs (**Figure 2E**); the network comprised 29 genes and 144 interacting mechanisms.

**Table 1:** Clinicopathological characteristics of glioma patients from the CGGA,TCGA, and GEO databases.

	CGGA-Training cohort	CGGA-Testing cohort	TCGA validation cohort	GSE4290
	N = 486	N = 484	N = 603	N = 176
Age				
<42	219	221	246	NA
≥42	267	262	357	NA
Gender				
Male	276	295	349	NA
Female	210	189	254	NA
Normal Tissue	NA	NA	NA	23
Grade				
II	136	134	213	45
III	160	162	238	31
IV	189	185	152	77
IDH				
Wild	207	214	224	NA
Mutation	252	248	373	NA
1p/19q				
Codel	94	105	149	NA
Non-codel	359	338	449	NA
MGMT				
Methylated	228	228	NA	NA
un-methylated	186	175	NA	NA
Status				
Dead	300	172	179	NA
Alive	186	312	424	NA
RiskScore				
Low	243	252	298	NA
High	243	232	305	NA

NA, Non available.

### Candidate Genes for the Focal Adhesion-Relevant Risk Signature

To identify the association with clinical information, we used 970 samples with available OS data and living status information from the CGGA database for the subsequent analysis. We divided 486 patient samples into the training cohort and 484 samples into the testing cohort. The candidate genes were analyzed using the LASSO regression analysis to exclude overlapping genes (**Figures 2F, G**), and multivariate Cox proportional risk regression analysis was performed (**Figure 2H**). Eight genes were obtained from the analysis: *COL1A2*, *COL4A1*, *ITGB4*, *MAPK10*, *PRKCB*, *PRKCG*, *RELN*, and *TNC. COL1A2*, *MAPK10*, and *PRKCB* genes were identified as low-risk genes, while *COL4A1*, *ITGB4*, *PRCKG*, *RELN*, and *TNC* were defined as high-risk for the OS in patients with glioma. The differential expression of each gene and immunochemistry images were obtained from online databases (**Supplementary Figures 1** and **2**).

A focal adhesion relevant prognostic signature was constructed using the following formula:


$$\begin{array}{c} \text{Risk score}=(-0.16036*\text{COL}1\text{A}2\text{ expression})+(0.321935*\text{COL}4\text{A}1\text{ expression})+(0.115143*\text{ITGB}4\text{ expression})\\ +\;(-0.18388*\text{MAPK}10\text{ expression})+(-0.20014*\text{PRKCB expression})+(0.191394*\text{PRKCG expression})\\ +\;(0.182231*\text{RELN expression})+(0.251663*\text{TNC expression}) \end{array}$$

Patients were divided into high- and low-risk groups according to the median of the risk score.

### Clinical Features of the Focal Adhesion-Related Differential Genes Signature in the Low- and High-Risk Groups

**Figure 3:**
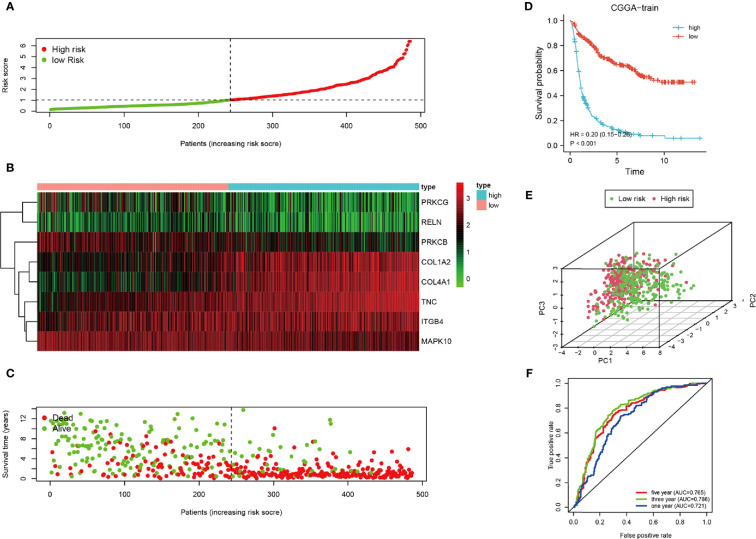
Analysis of the CGGA training cohort. **(A)** Cutoff of low-and high-risk patients **(B)** Heatmap of expression of candidate genes. **(C)** Survival status of low-and high-risk patients. **(D)** Survival analysis of low- and high-risk patients. **(E)** PCA of low-and high-risk patients. **(F)** ROC of 1-, 3-, and 5-year OS.

To clarify the relationship between the prognosis and FADGs signature, we analyzed the clinical information of 486 samples in the training cohort, 484 samples in the testing cohort for internal authentication, and 603 samples in the TCGA database for external validation. The results of the analysis of the training cohort are shown in **Figure 3**. Low- and high-risk groups were stratified according to the median of the risk score (**Figure 3A**). Expression levels of the eight genes from the training cohort are shown in **Figure 3B**. Survival time of high-risk patients tended to be worse than low-risk patients (**Figure 3C**). The survival probability of the high-risk score group was significantly lower than that of the low-risk group [hazard ratio (HR) = 0.02, 95% confidence interval (CI) = 0.15–0.25, P < 0.001, C-index = 0.685) (**Figure 3D**). Patients could be stratified into two groups according to our risk score (**Figure 3E**). The areas under the curve for the 1-, 3-, and 5-year survival were 0.721, 0.786, and 0.765, respectively (**Figure 3F**).

Univariate regression analysis showed that this signature could predict the prognosis of patients with glioma and resulted to be an independent prognostic factor from the multivariate regression analysis (**Supplementary Figures 3A, B**). In summary, our signature was a sensitive prognostic model for the risk stratification of patients with glioma.

**Figure 4:**
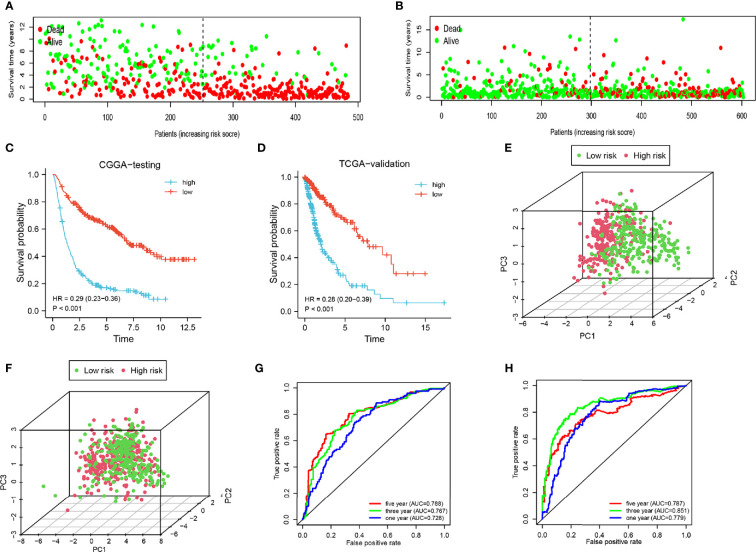
Analysis of the testing cohort and validation cohort. **(A, B)** Survival status of low-and high-risk patients in the CGGA testing and TCGA cohorts. **(C, D)** Survival analysis of low- and high-risk patients in the CGGA testing and TCGA cohorts. **(E, F)** PCA of low-and high-risk patients in the CGGA testing and TCGA cohorts. **(G–H)** ROC of 1-, 3-, and 5-year in the CGGA testing and TCGA cohorts.

To increase the credibility of our model, we performed the same analysis using the CGGA internal authentication cohort and TCGA external validation cohort. Survival time of patients with high-risk scores tended to be shorter than patients with low-risk scores (**Figures 4A, B**). The survival probability of the high-risk group was significantly lower in both cohorts (CGGA: HR = 0.29, 95% CI = 0.23–0.36, P < 0.001, C-index = 0.661); (TCGA: HR = 0.28, 95% CI = 0.20–0.39, P < 0.001, C-index = 0.660) (**Figures 4C, D**). Patients from the CGGA testing and TCGA groups could be stratified into two subgroups distinctly by PCA analysis (**Figures 4E, F**). ROC curves were plotted and the areas under the curve for the 1-, 3-, and 5-year survival in the CGGA testing cohort were 0.728, 0.767, and 0.788, respectively, (**Figure 4G**) and 0.779, 0.851, and 0.787, respectively, in the TCGA validation cohort (**Figure 4H**). Univariate regression analysis and multivariate regression analysis demonstrated the 8-gene signature was an acute prognostic factor (**Supplementary Figures 3C, D**). The same with the CGGA training cohort, our signature was a sensitive prognostic model.

**Figure 5:**
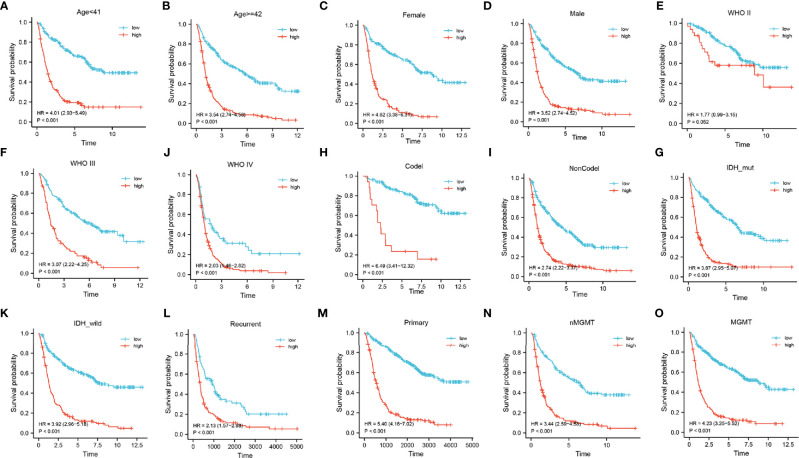
**(A–O)** Stratified survival analysis of low- and high-risk patients in the CGGA database, by age, gender, grade, 1p19q codeletion, IDH mutant, PRS, and MGMT status.

Next, a stratified analysis was performed using both the CGGA and TCGA databases (**Figure 5** and **Supplementary Figure 4** **)**. Patients in the CGGA were stratified by grade, gender, age, *IDH* mutational status, 1p19q co-deletion status, *MGMT* methylation status, and PRS status. And in the TCGA cohort were stratified by grade, sex, age, *IDH* mutational status, and 1p19q co-deletion status. According to the FADGs signature, the survival probability of patients in the high-risk group was significantly lower than that in the low-risk group (P < 0.001), except for the WHO II subgroup (P = 0.052). These findings indicated that the FADG signature played a certain role in predicting the prognosis of glioma patients.

### Gene Set Enrichment Analysis

**Figure 6:**
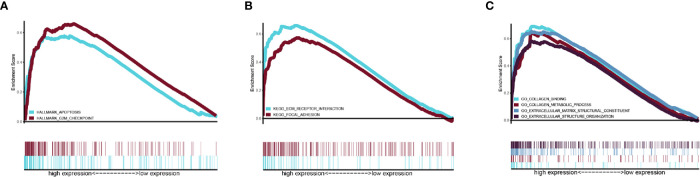
Related pathway were analyzed by GSEA. **(A)** Hallmark analysis of our signature **(B)** KEGG analysis of our signature. **(C)** GO analysis of our gene signature.

Gene set Enrichment analysis was programmed between the high- and low-risk groups with a threshold of NES > 1 and FDR q-val < 0.05. In the Hallmark analysis, the APOPTOSIS and G2M CHECK POINT pathway was enriched (**Figure 6A**). In the KEGG analysis, the KEGG_ECM_RECEPTOR_INTERACTION pathway was enriched (**Figure 6B**), while GO analysis revealed that the GO_EXTRACELLULAR_STRUCTURE_ORGANIZATION, GO_COLLAGEN_METABOLIC_PROCESS, GO_COLLAGEN_BINDING, and GO_EXTRACELLULAR_MATRIX_STRUCTURAL_CONSTITUENT components were enriched (**Figure 6C**).

### Relationship Between Radiation Response and the Focal Adhesion-Related Differential Gene Signature

**Figure 7:**
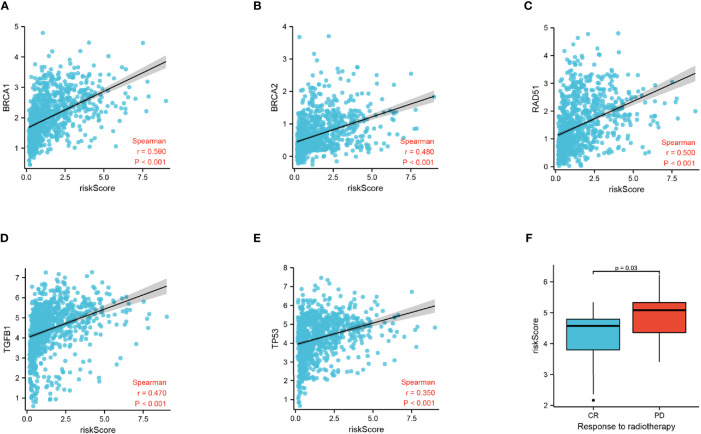
Correlation of risk score and radiation response genes. **(A)** Correlation of risk score and BRCA1. **(B)** Correlation of risk score and BRCA2. **(C)** Correlation of risk score and RAD51. **(D)** Correlation of risk score and TGFB1. **(E)** Correlation of risk score and TP53. **(F)** Risk score of the CR and PD groups.

To discover the relationship between the radiation response and our FADG signature, we performed a correlation analysis between genes that related to DNA repair and our FADG signature. Our signature is positively correlated with BRCA1 (r = 0.590, P < 0.001) (**Figure 7A**), BRCA2 (r = 0.480, P < 0.001) (**Figure 7B**), RAD51 (r = 0.500, P < 0.001) (**Figure 7C**), TGFB1 (r = 0.470, P < 0.001) (**Figure 7D**), and TP53 (r = 0.350, P < 0.001) (**Figure 7E**). The risk scores of patients in the CR group were significantly lower than in the PD group (P = 0.028) (**Figure 7F**).

### Relationship Between the Tumor Immune Microenvironment and the Focal Adhesion-Related Differential Gene Signature

**Figure 8:**
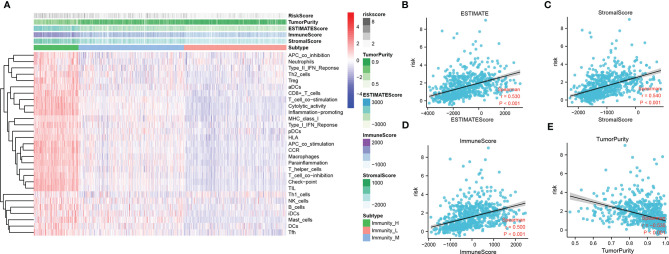
Relationship between immune cell infiltration and risk score. **(A)** Heatmap of ssGSEA and correlation between risk and ESTIMATE, immune, and stromal scores, and tumor purity. **(B)** Correlation of risk and ESTIMATE scores. **(C)** Correlation of risk and stromal scores. **(D)** Correlation of risk and immune scores. **(E)** Correlation of risk and tumor purity scores.

**Figure 9:**
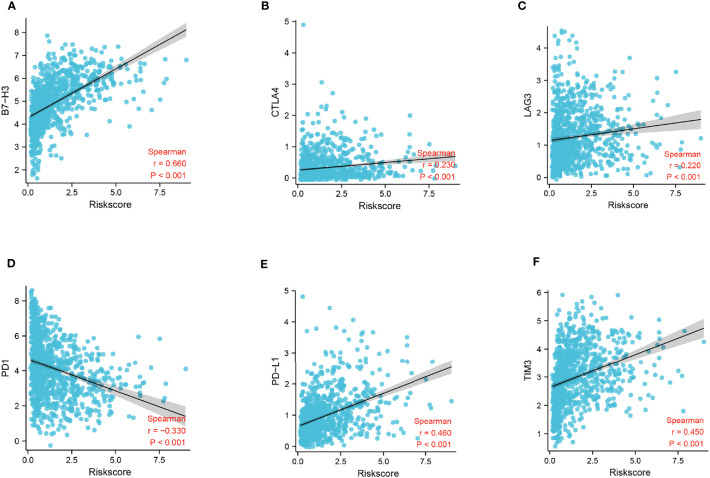
Correlation analysis of risk score and immune checkpoints. **(A)** Correlation analysis of risk score and B7-H3 levels. **(B)** Correlation analysis of risk score and CTLA4 levels. **(C)** Correlation analysis of risk score and LAG3 levels. **(D)** Correlation analysis of risk score and gene PD-1 levels. **(E)** Correlation analysis of risk score and PD-L1 levels. **(F)** Correlation analysis of risk score and gene TIM3 levels.

To identify the potential relationship between immune cell infiltration and our FADG signature, we performed ESTIMATE analysis and CIBERSORT analysis. From the ESTIMATE analysis, patients with high-risk scores tended to gather in the IMMUNITY_H group. High-risk score group was positively correlated with the ESTIMATE, immune, and stromal scores and conversely, negatively correlated with tumor purity (**Figure 8A**). To verify whether there was a statistically significant association, correlation analysis was performed (**Figures 8B–E**). CIBERSORT was performed to explore the status of immune cell infiltration. Bar plots showed the proportion of immunocytes in each patient (**Supplementary Figure 5A**). Furthermore, levels of resting memory CD4+T cells (P = 0.003), follicular helper T cells (P < 0.001), regulatory T cells (Tregs)(P = 0.01), gamma delta T cells (P = 0.009), and M0 macrophages (P < 0.001) were significantly positively related with the risk score, while levels of naïve CD4+T cells (P < 0.001), activated NK cells (P = 0.014), and monocytes (P < 0.001) were significantly negatively related with the risk score (**Supplementary Figure 5B**). The correlation heatmap showed the correlation between the risk score and immunocyte levels (**Supplementary Figure 5C**). Finally, a correlation analysis between our signature and immune check points was conducted. Our signature positively correlated with levels of *B7-H3* (r = 0.660, P < 0.001), *CTLA4* (r = 0.230, P < 0.001), *LAG3* (r = 0.220, P < 0.001), *PD-L1* (r = 0.460, P < 0.001), and *TIM3* (r = 0.450, P < 0.001), and negatively correlated with *PD-1* (r = -0.330, P < 0.001) (**Figures 9A–F**). These results showed that our model was closely correlated with immunocyte infiltration and immune check points.

### Construction and Evaluation of the Nomogram

**Figure 10:**
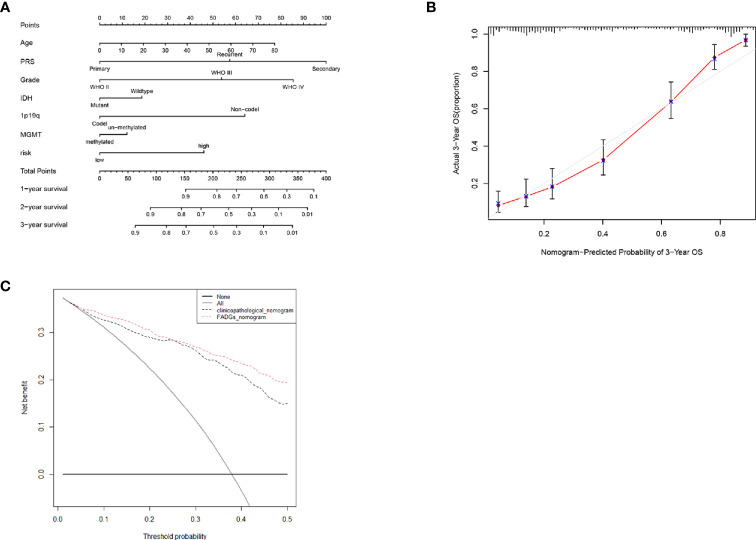
Nomogram was constructed with the risk score and clinical characteristics **(A)** Nomogram of the clinical features and risk score. **(B)** Calibration curve of the actual 3-year OS. **(C)** DCA analysis of the FADGs signature.

To create a sensitive predictive model of prognosis, a nomogram was constructed using data from the CGGA cohort. Each clinical feature and the relative risk score were considered to calculate the total point score. A probability of the 1-, 2-, and 3-year OS was reflected by the total points shown in **Figure 10A**. To estimate the validity of the nomogram, calibration plots were programmed. The calibration plots revealed that the nomogram had a good stability for predicting the actual 3-year OS (**Figure 10B**). Decision curve analysis (DCA) was used to calculate the incremental value in adding the FADG score to the nomogram. Obviously, the prediction level of adding the FADG score to the nomogram was significantly higher than that of the normal (**Figure 10C** **)**. Based on the above analysis, our focal adhesion-relevant signature score could predict the OS of patients with glioma.

## Discussion

Glioma represents 81% of all malignant craniocerebral tumors (12). Glioma, and especially GBM, is characterized by highly malignant disease, death, and recurrence rates, and accounts for 50% of these malignancies (13). Some molecular features have been adopted in the clinical practice, for example, *IDH* mutational status and *MGMT* methylation are considered markers of a better prognosis. Although advances in immunotherapy have improved outcomes for some cancers, there is still a significant challenge for the optimal management of glioma (14).

Focal adhesion is at the center of signaling pathways crucial for tumor development (15). Focal adhesion molecules act as binding sites within the cell and matrix to allow integrin binding and induction of cell migration (16, 17). Cells extend protrusions to migrate and transmembrane receptors, which are the stabilizer of these cellular protrusions, anchor to the actin cytoskeleton *via* FA complexes to provide cells the power to undergo migration (18). Leukocytes migrate to the peripheral tissue to perform immune surveillance functions (18). In the TME of glioma, glioma-associated microglia or macrophages and myeloid-derived suppressor cells represent the most infiltrated cell types, and their levels have been shown to be negatively correlated with the prognosis. Furthermore, myeloid-derived suppressor cells can inhibit the cytotoxic responses mediated by NK cells (19).

We identified a FADG signature by mining databases, which included *COL1A2*, *COL4A1*, *ITGB4*, *MAPK10*, *PRKCB*, *PRKCG*, *RELN*, and *TNC*. Some studies found that COL1A2 was a crucial gene regulating cell migration by the cytoskeleton. Silencing of *COL1A2* inhibited gastric cancer progression (20). Wang et al. demonstrated that *COL4A1* promoted metastasis in hepatocellular carcinoma (21). *ITGB4* maybe an early detector and a prognostic element for colorectal cancer (22). *MAPK10*, also known as *JNK3*, suppressed the expression of *JNK3* and enhanced the toxicity of paclitaxel in head and neck cancer cells (23). The activation of *PRKCB* accelerated the mitochondrial accumulation and the redox response to enhance signaling transduction pathways in cancer cells (24). Polymorphism of SNP rs454006 in *PRKCG* was demonstrated to increase the risk of patients with osteosarcoma (25). *RELN* regulated the migration of glioma cells, and activation of the RELN-related pathway could suppress the proliferation of GBM cells (26). *TNC* was found to support the aggressiveness of breast cancer cells and promote micrometastases (27). In our study, patient samples were scored using our FADGs signature, and was able to stratify patients into high- and low-risk groups according to the median risk score. We used the CGGA training cohort to conduct a survival analysis, ROC curves, PCA, stratification analysis, and multivariate and univariate regression analysis. Our findings demonstrated that the identified gene signature was a valuable parameter for predicting the prognosis. We achieved similar results using an internal authentication cohort and external validation cohort.

We performed gene set enrichment analysis to explore the mechanisms about the FADG signature. Our signature was enriched in apoptosis and G2M check point which is related with the cell cycle from the results. Cell cycle is the decisive factor of radiation sensitivity. Cells in the G2-M phase is most sensitive to ionizing radiation (28). Then, we found that our signature was closely related with genes that related with DNA repair. Patients after radiation with *BRCA1* mutation had a better prognosis was identified by Kan (29). *BRCA2*-mediated cell survival suffered from radiation (30). Inhibition of *RAD51* enhanced the radiation sensitivity of glioma stem cells (31). Radiation could activate the *TGF-beta1* signaling pathway and induce radiation resistance (32). *P53* enhanced the process of DNA repair and lead to the failure of radiation therapy (33). The risk score of patients in the complete remission group was significantly lower than those with progression disease. In short, we considered that the FADG signature could predict the response of radiation therapy.

Immune cells exercised their immune surveillance function by cell migration. The tumor immune microenvironment is complicated in glioma. Glioma-related myeloid cells exert a significant effect to promote the aggressiveness of glioma cells (11). To explore the brain immune microenvironment of patients with glioma, we conducted ssGSEA and CIBERSORT. Correlation heatmap showed that the ESTIMATE score, immune score, and stromal score increased as the risk score increased, and conversely, tumor purity decreased. We performed a correlation analysis to validate the results. The risk score is positively correlated with the ESTIMATE, immune, and stromal scores, and negatively correlated with tumor purity. These results indicated that in the tumor immune microenvironment, the infiltration of immune cells was higher in high-risk glioma patients. Our findings were consistent with previous studies indicating that the degree of tumor infiltration of immune cells increased with an increasing grade (34). Our results from the CIBERSORT analysis confirmed the notion that glioma cells were enriched by the secretion of immune cells, such as leukocytes, CD4+T cells, and Tregs (35). In the tumor immune microenvironment of glioma, polarization of M2 macrophage has been reported to lead to the malignant biological behavior of glioma cells (36). High infiltration of regulatory T cells was strongly associated with a poor prognosis (36). In this study, we determined that there were more M2 macrophages and regulatory T cells in the high-risk group than other cell types. The ECM played an important role in cell migration and immune response (37). Furthermore, we found that several pathways relevant to the ECM were enriched. These findings provided support that our signature had a strong connection with immune cell infiltration.

Immune checkpoints inhibitors have shown a surprising efficacy in many malignancies, such as lung cancers, gastric cancers, breast cancers, and glioma (2, 6, 38, 39). B7-H3 is a molecule in the B7 family which has been reported to be overexpressed in non-small cell lung cancer, and positively correlated with lymph node metastasis and TNM stage. In addition, B7-H3 has been associated with Tregs levels (40). Cytotoxic T Lymphocyte antigen 4 (CTLA-4) has been shown to inhibit the function of T cell activation, suppressing the immune system function (41). Sustained co-expression of lymphocyte activation gene-3 (LAG3) on T cells also impaired the function of T cells leading to dysfunction of the cellular immunity (42). Programmed cell death protein-1 (PD-1) binds with programmed cell death-1 ligand 1 (PD-L1) and inhibits the activation of T cells, resulting in dysfunction of the immune surveillance (43). High expression of T cell immunoglobulin and mucin domain-containing protein 3 (TIM3) has been positively correlated with a shorter OS, and its co-expression with PD-1 is associated with a poor prognosis (34). Our risk score was positively correlated with *B7-H3*, *CTLA-4*, *LAG3*, *PD-L1*, and *TIM3* expression, while it was negatively correlated with *PD-1* expression. We propose that our signature may represent a new approach to guide clinical treatment by immunotherapy.

Nonetheless, our study presented some limitations. First, the clinical features obtained from the TCGA database were incomplete and lacked the *MGMT* methylation status and PRS types. Second, our study requires experimental validation both *in vivo* and *in vitro*. Third, the CGGA database only consisted of Chinese patient samples.

## Conclusion

Our focal adhesion relevant signature combined with the clinical features may predict patient prognosis more accurately and may represent a novel approach to the management of immunotherapy treatment for patients with glioma.

## Data Availability Statement

The datasets presented in this study can be found in online repositories. The names of the repository/repositories and accession number(s) can be found in the article/**Supplementary Material**.

## Ethics Statement

Ethical review and approval were not required for the study on human participants in accordance with the local legislation and institutional requirements. Written informed consent for participation was not required for this study in accordance with the national legislation and the institutional requirements.

## Author Contributions

XX was responsible for the overall design of this study. GW, HL, and WW analyzed the data and edited the manuscript. JP contributed the study guidance of R software. HZ examined the data analysis. XH and LS provided R language modification. HZ revised the images and tables of this article. LH revised the discussion of the article. LH contributed the study design. All authors contributed to the article and approved the submitted versions.

## Funding

This study was supported by the Key R&D program of Hebei Province (19277737D).

## Conflict of Interest

The authors declare that the research was conducted in the absence of any commercial or financial relationships that could be construed as a potential conflict of interest.

## Publisher’s Note

All claims expressed in this article are solely those of the authors and do not necessarily represent those of their affiliated organizations, or those of the publisher, the editors and the reviewers. Any product that may be evaluated in this article, or claim that may be made by its manufacturer, is not guaranteed or endorsed by the publisher.

## Supplementary Material

Supplementary Figure 1 **(A–H)** Differential expression of 8 candidate genes of the FADG signature in the LGG and normal brain tissue. **(I-P)** Differential expression of 8 candidate genes of the FADG signature in the GBM and normal brain tissue.

Supplementary Figure 2 **(A–H)** Immunochemistry of 8 candidate genes of the FADG signature.

Supplementary Figure 3 **(A, D)** Univariate and multivariate Cox regression analysis of clinical features and the FADG signature in the CGGA training cohort. **(B, E)** Univariate and multivariate Cox regression analysis of clinical features and the FADG signature in the CGGA testing cohort. **(C, F)** Univariate and multivariate Cox regression analysis of clinical features and the FADG signature in the TCGA validation cohort.

Supplementary Figure 4 **(A–K)** Stratified survival analysis of low- and high-risk patients in TCGA database.

Supplementary Figure 5 **(A)** Proportion of immunocytes in the training cohort. **(B)** Differences between low-and high- risk groups in terms of immunocyte infiltration. **(C)** Correlation heatmap of risk score and immunocyte infiltration.

## Abbreviations

GEO, Gene Expression Omnibus; TCGA, The Cancer Genome Atlas; CGGA, Chinese Glioma Genome Atlas; FADG, Focal Adhesion-related Differential Gene; TME, Tumor Microenvironment; TTF, Tumor Treatment Fields; NCCN, National Comprehensive Cancer Network; OS, Overall Survival; WHO, World Health Organization; GBM, Glioblastoma Multiforme; DEG, Differentially Expressed Genes; PPI, Protein-protein Interaction; CR, Complete Remission; PD, Progression Disease.
