# Supplementary figures and images for: A Focal Adhesion-Related Gene Signature Predicts Prognosis in Glioma and Correlates With Radiation Response and Immune Microenvironment
Source: Front Oncol. 2021 Sep 22;11:698278. doi: 10.3389/fonc.2021.698278 (PMC8493301; doi:10.3389/fonc.2021.698278)

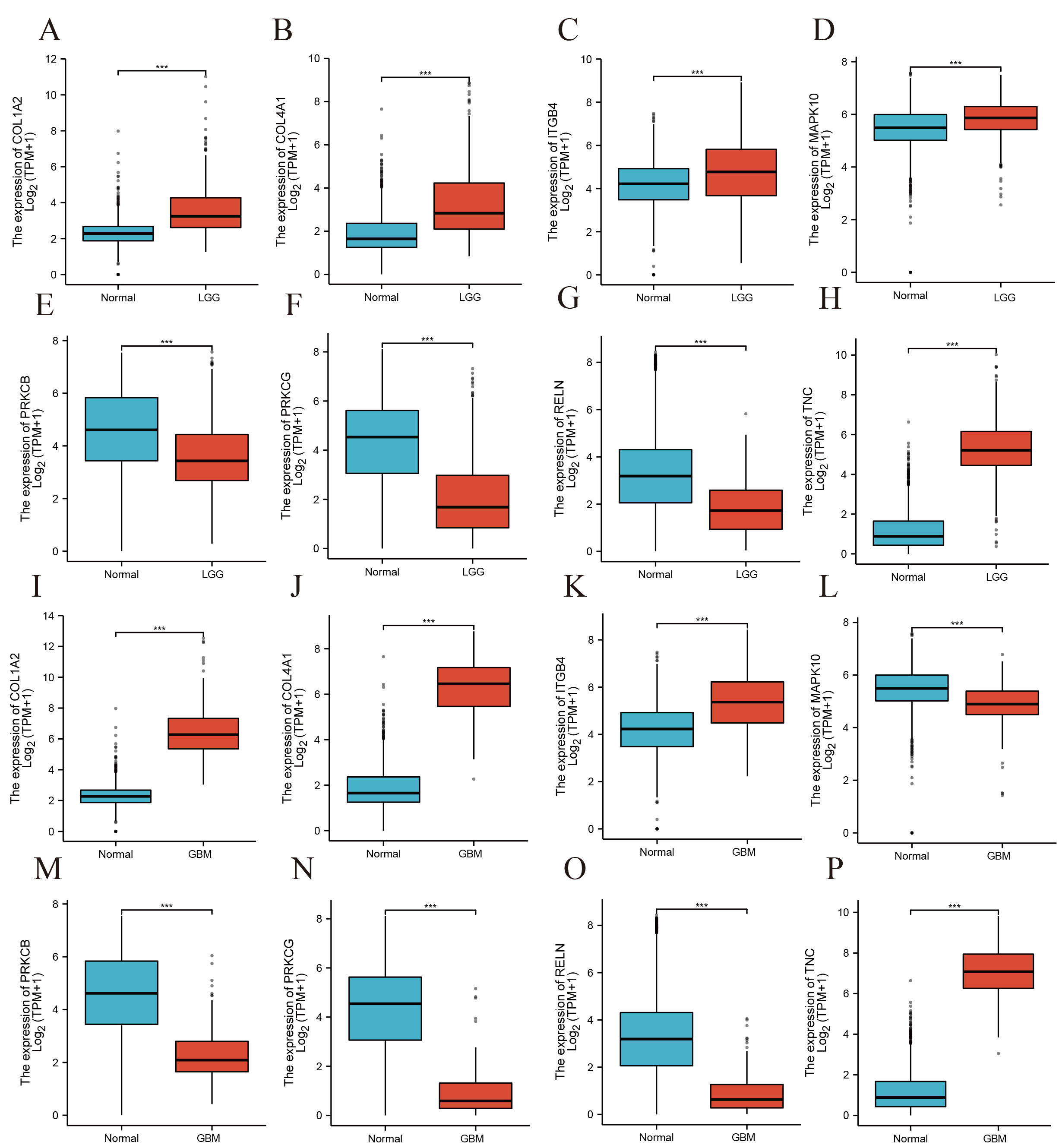

Supplement: Supplementary Figure 1 — (A–H) Differential expression of 8 candidate genes of the FADG signature in the LGG and normal brain tissue. (I-P) Differential expression of 8 candidate genes of the FADG signature in the GBM and normal brain tissue. [file Image_1.tif]

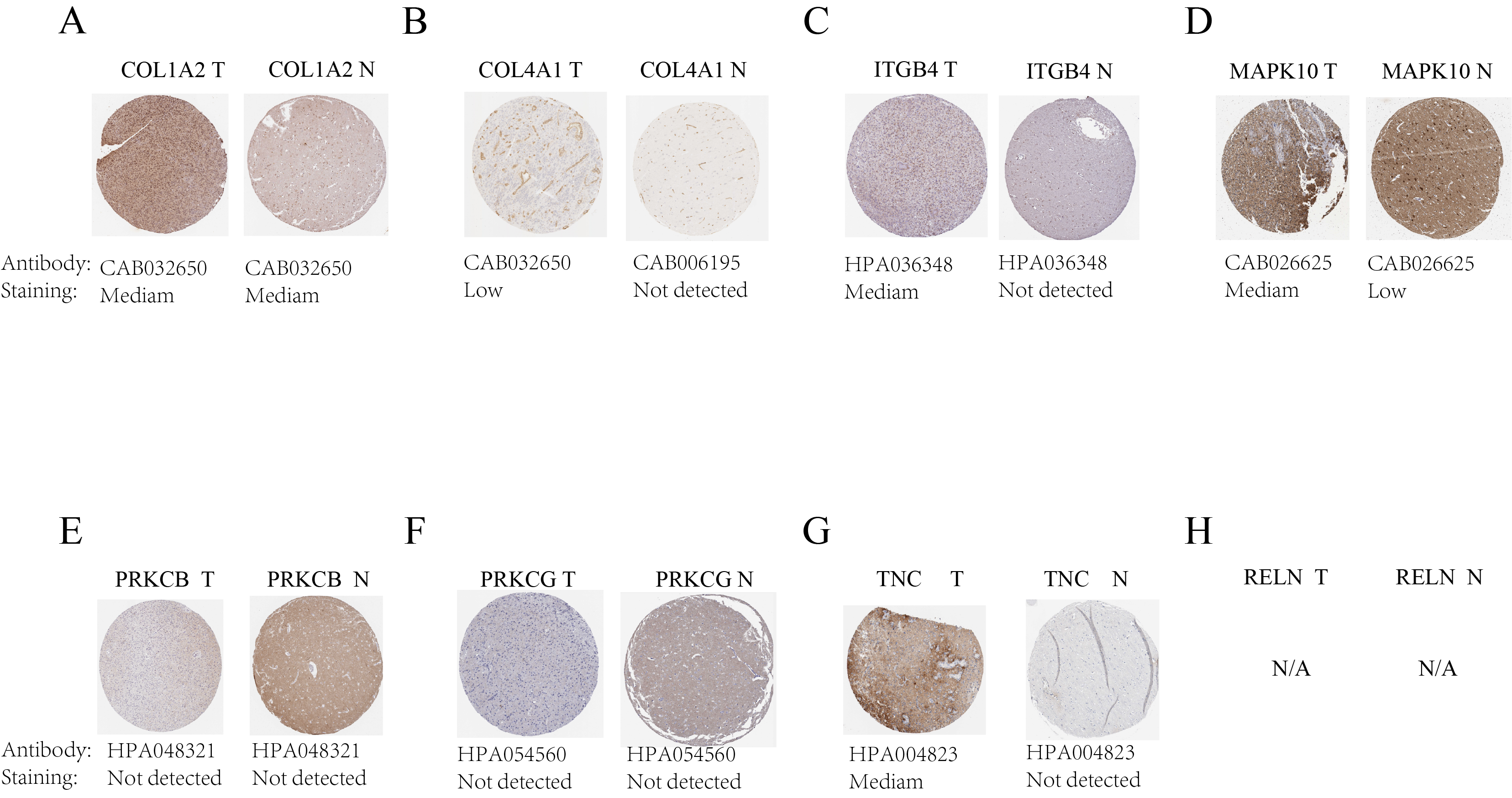

Supplement: Supplementary Figure 2 — (A–H) Immunochemistry of 8 candidate genes of the FADG signature. [file Image_2.tif]

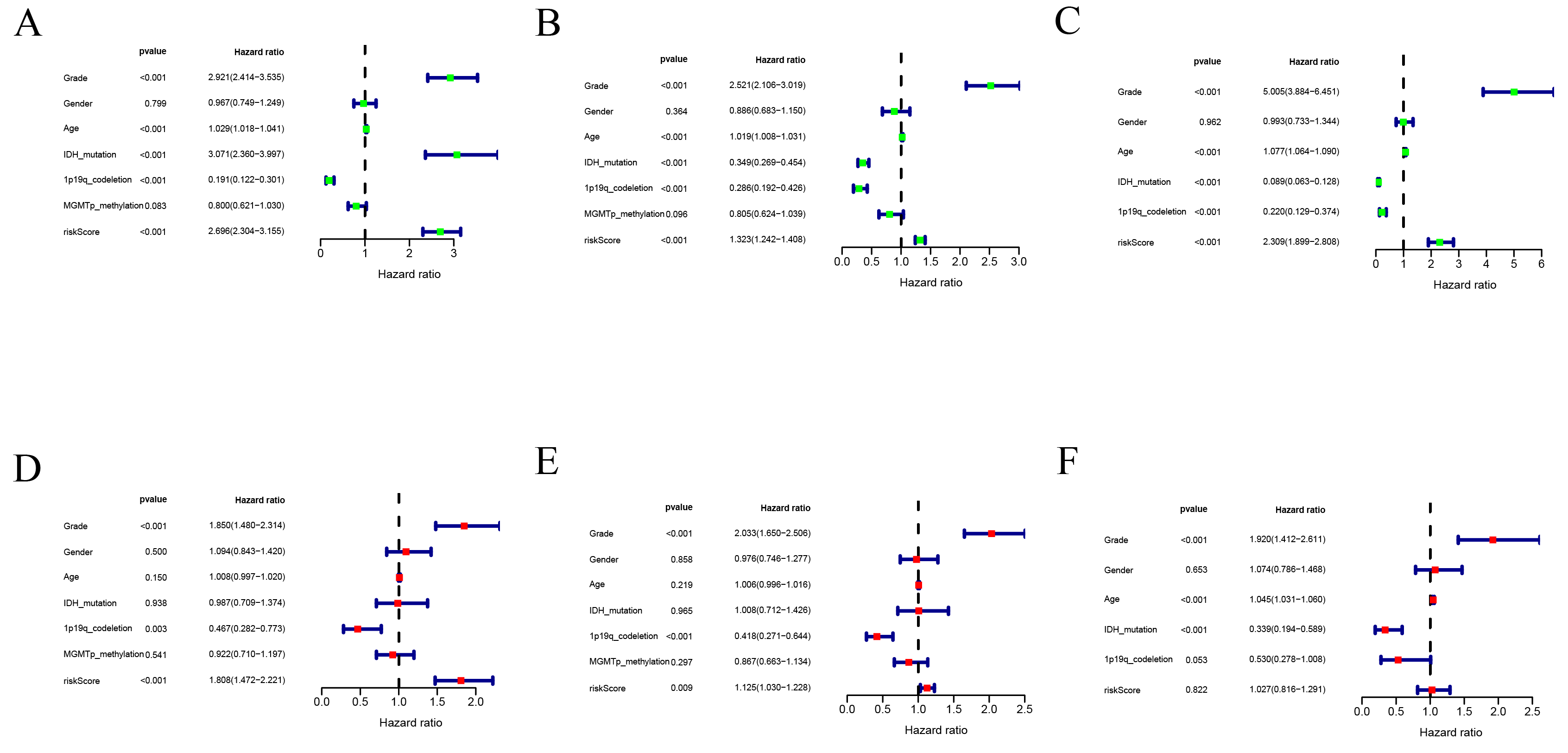

Supplement: Supplementary Figure 3 — (A, D) Univariate and multivariate Cox regression analysis of clinical features and the FADG signature in the CGGA training cohort. (B, E) Univariate and multivariate Cox regression analysis of clinical features and the FADG signature in the CGGA testing cohort. (C, F) Univariate and multivariate Cox regression analysis of clinical features and the FADG signature in the TCGA validation cohort. [file Image_3.tif]

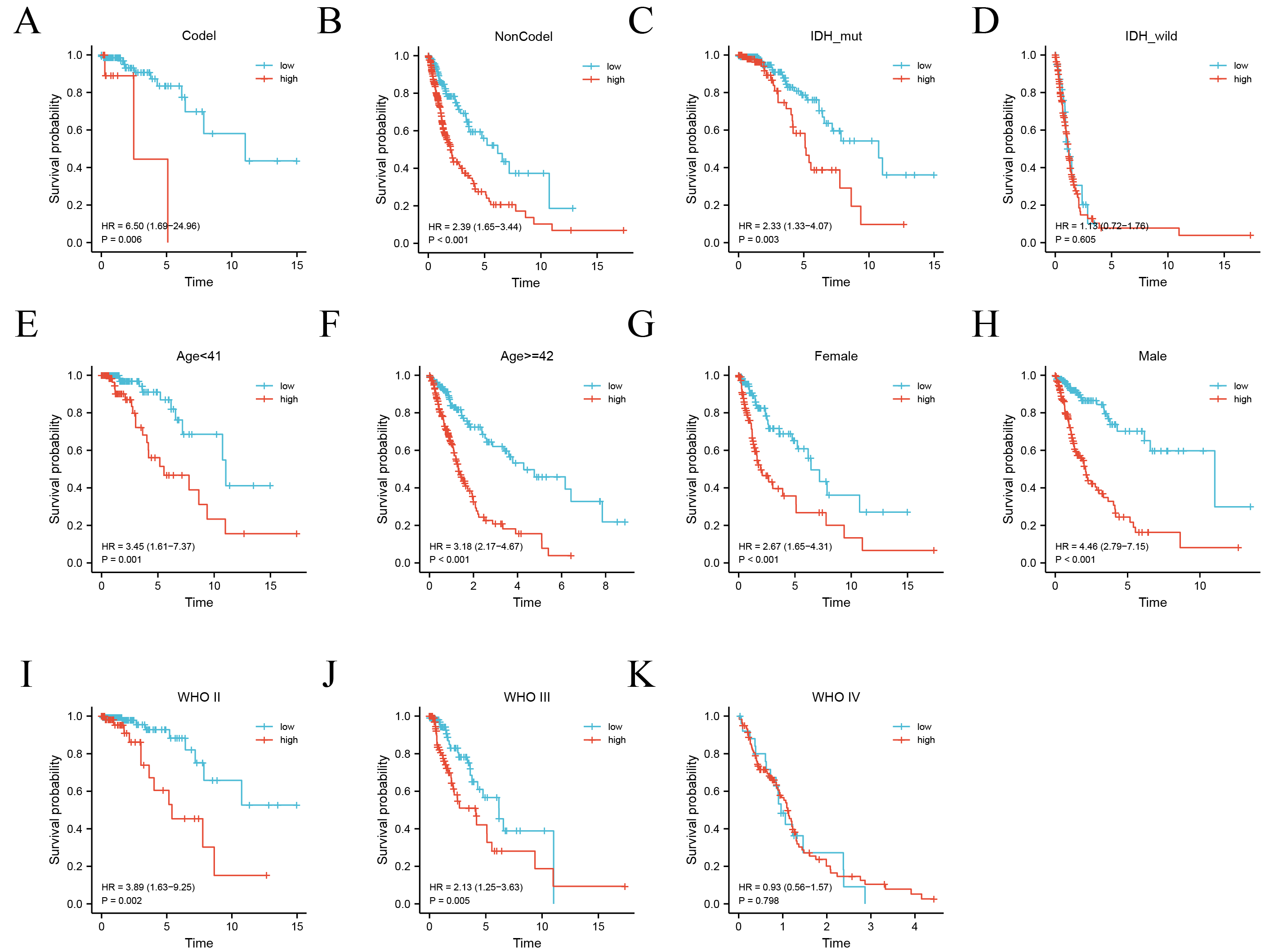

Supplement: Supplementary Figure 4 — (A–K) Stratified survival analysis of low- and high-risk patients in TCGA database. [file Image_4.tif]

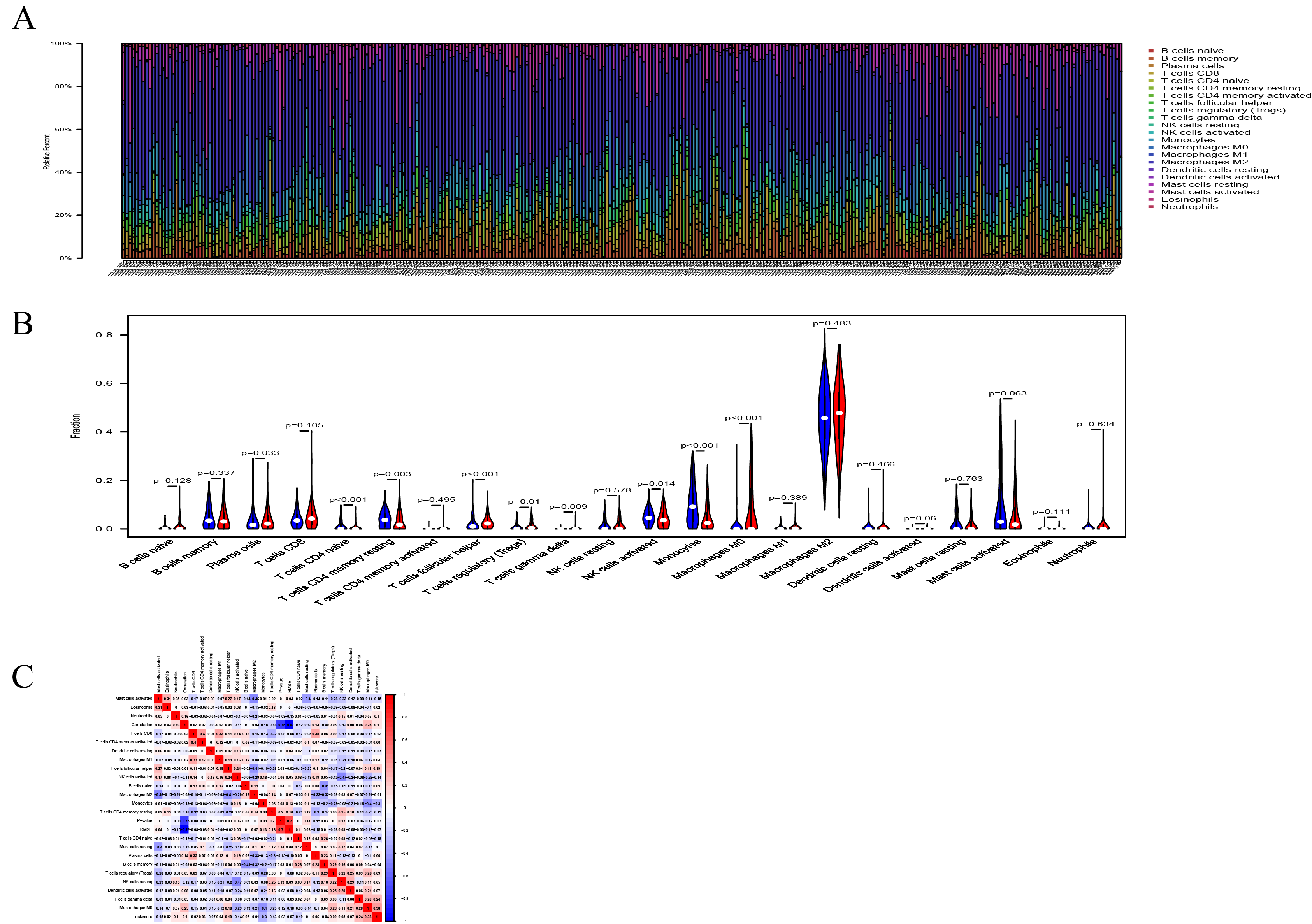

Supplement: Supplementary Figure 5 — (A) Proportion of immunocytes in the training cohort. (B) Differences between low-and high- risk groups in terms of immunocyte infiltration. (C) Correlation heatmap of risk score and immunocyte infiltration. [file Image_5.tif]
