# Supplementary material for: A Focal Adhesion-Related Gene Signature Predicts Prognosis in Glioma and Correlates With Radiation Response and Immune Microenvironment
Source: Front Oncol. 2021 Sep 22;11:698278. doi: 10.3389/fonc.2021.698278 (PMC8493301; doi:10.3389/fonc.2021.698278)
Supplement: Supplementary file 6 [file Table_1.docx]

Supplementary Table1: Focal Adhesion relevant genes

| ACTB | CHAD | EGFR | ITGA5 | LAMC2 | PAK6 | PRKCA | SRC |
| --- | --- | --- | --- | --- | --- | --- | --- |
| ACTG1 | COL11A1 | ELK1 | ITGA6 | LAMC3 | PARVA | PRKCB | THBS1 |
| ACTN1 | COL11A2 | ERBB2 | ITGA7 | MAP2K1 | PARVB | PRKCG | THBS2 |
| ACTN2 | COL1A1 | FLNA | ITGA8 | MAPK1 | PARVG | PTEN | THBS3 |
| ACTN3 | COL1A2 | FLNB | ITGA9 | MAPK10 | PDGFA | PTK2 | THBS4 |
| ACTN4 | COL2A1 | FLNC | ITGAV | MAPK3 | PDGFB | PXN | TLN1 |
| AKT1 | COL3A1 | FLT1 | ITGB1 | MAPK8 | PDGFC | RAC1 | TLN2 |
| AKT2 | COL4A1 | FLT4 | ITGB3 | MAPK9 | PDGFD | RAC2 | TNC |
| AKT3 | COL4A2 | FN1 | ITGB4 | MET | PDGFRA | RAC3 | TNN |
| ARHGAP35 | COL4A4 | FYN | ITGB5 | MYL10 | PDGFRB | RAF1 | TNR |
| ARHGAP5 | COL4A6 | GRB2 | ITGB6 | MYL12A | PDPK1 | RAP1A | TNXB |
| BAD | COL5A1 | GSK3B | ITGB7 | MYL12B | PGF | RAP1B | VASP |
| BCAR1 | COL5A2 | HGF | ITGB8 | MYL2 | PIK3CA | RAPGEF1 | VAV1 |
| BCL2 | COL5A3 | HRAS | JUN | MYL5 | PIK3CB | RASGRF1 | VAV2 |
| BIRC2 | COL6A1 | IBSP | KDR | MYL7 | PIK3CD | RELN | VAV3 |
| BIRC3 | COL6A2 | IGF1 | LAMA1 | MYL9 | PIK3CG | RHOA | VCL |
| BRAF | COL6A3 | IGF1R | LAMA2 | MYLK | PIK3R1 | ROCK1 | VEGFA |
| CAPN2 | COL6A6 | ILK | LAMA3 | MYLK2 | PIK3R2 | ROCK2 | VEGFB |
| CAV1 | COMP | ITGA1 | LAMA4 | MYLK3 | PIK3R3 | SHC1 | VEGFC |
| CAV2 | CRK | ITGA10 | LAMA5 | MYLPF | PIK3R5 | SHC2 | VEGFD |
| CAV3 | CRKL | ITGA11 | LAMB1 | PAK1 | PIP5K1C | SHC3 | VTN |
| CCND1 | CTNNB1 | ITGA2 | LAMB2 | PAK2 | PPP1CA | SHC4 | VWF |
| CCND2 | DIAPH1 | ITGA2B | LAMB3 | PAK3 | PPP1CB | SOS1 | XIAP |
| CCND3 | DOCK1 | ITGA3 | LAMB4 | PAK4 | PPP1CC | SOS2 | ZYX |
| CDC42 | EGF | ITGA4 | LAMC1 | PAK5 | PPP1R12A | SPP1 |  |
